# Complete mitochondrial DNA sequence of the endangered fish (*Bahaba taipingensis*): Mitogenome characterization and phylogenetic implications

**DOI:** 10.3897/zookeys.546.5964

**Published:** 2015-12-16

**Authors:** Linlin Zhao, Tianxiang Gao, Weihua Lu

**Affiliations:** 1 The First Institute of Oceanography, SOA, Qingdao, Shandong, 266003, P.R. China; 2 Fishery College, Zhejiang Ocean University, Zhoushan, Zhejiang, 316000, P.R. China; 3 Dongguan Bahaba Natural Conservation and Management Station, Dongguan, Guangdong, 523002 P.R. China

**Keywords:** *Bahaba taipingensis*, Sciaenidae, mitochondrial genome, control region, phylogenetic analysis

## Abstract

To understand the systematic status of *Bahaba taipingensis* within Sciaenidae, the complete mitochondrial genome (mitogenome) sequence of Chinese bahaba has recently been determined by long PCR and primer walking methods. The complete mitochondrial genome is 16500 bp in length and contains 37 mitochondrial genes (13 protein-coding genes, 2 ribosomal RNA genes and 22 transfer RNA genes) as well as a control region (CR) as other bony fishes. Within the control region, we identified the extended termination associated sequence domain (ETAS), the central conserved sequence block domain (CSB-D, SCB-E and CSB-F) and the conserved sequence block domain (CSB-1, CSB-2 and CSB-3). Phylogenetic analyses revealed that *Bahaba taipingensis* is more closely related to Pseudosciaeniae than Argyrosominae and Sciaeninae. Additionally, *Bahaba taipingensis* is the sister taxon of *Miichthys miiuy*, and those two are sister to *Collichthys* plus *Larimichthys*.

extended termination associated sequence domain

## Introduction

The complete mitochondrial DNA (mtDNA) sequence of vertebrates is a circular molecule with a length of 16-19 kb that includes 37 genes containing 13 protein-coding genes, 2 ribosomal RNA (rRNA) genes, 22 transfer RNA (tRNA) genes, and a control region (CR) (Anderson et al. 1981; Boore et al. 1999). The mitochondrial genome is frequently used for phylogenetic studies and population genetic analyses, due to its compact gene organization, fast evolutionary rate, maternal inheritance and lack of genetic recombination (Miya et al. 2003; Inoue et al. 2009). In recent years, complete mitochondrial DNA sequences have been widely used to reconstruct the phylogeny of higher-level taxa (Jondeung et al. 2007; Wang et al. 2008; Yang et al. 2010).

The family Sciaenidae in the order Perciformes is widely distributed throughout the world with approximately 70 genera and 300 species (Nelson 2006). Fishes of this family are popularly known as croakers and drums because of the ability using muscles associated with gas bladder to produce sound. In China, the family comprises 13 genera with about 37 species, and can be divided into seven subfamilies: Johniinae, Megalonibinae, Bahabinae, Sciaeninae, Otolithinae, Argyrosominae, Pseudosciaeniae (Zhu et al. 1963; Cheng et al. 1987; Tetsuji et al. 2000). The Chinese bahaba, *Bahaba taipingensis*, is one of the largest croakers and has a limited geographical distribution from Zhoushan Island southwards to the Pearl River (Zhu et al. 1963; Lu et al. 2002). Over the past years, its stock has been declining due to heavy catch pressure and environmental degradation, therefore it is defined as National Class II Protected Animals of China and Critically Endangered by the IUCN. There have been a few reports on the general ecology of this specie covering resources, biology, and otolith morphology (Lu et al. 2002; Ye et al. 2001; Ou et al. 2011). Additionally, the phylogenetic relationships of Sciaenidae have been investigated by means of molecular markers (Meng et al. 2004; Chen et al. 2007; Liu et al. 2010; Cheng et al. 2010), but only one study included *Bahaba taipingensis* (He et al. 2012), which revealed that *Bahaba taipingensis* is closely related to Pseudosciaeniae.

In this study, we sequenced the complete mtDNA sequence of *Bahaba taipingensis* for the first time and analyzed its genomic structure. Additionally, we conducted phylogenetic analyses based on the mitochondrial sequence data with the purpose of investigating the phylogenetic position of *Bahaba taipingensis* within the family Sciaenidae. The information reported in this article will facilitate further investigations of phylogenetic relationships of species in the Sciaenidae.

## Materials and methods

### Sample collection and DNA extraction

The sample of *Bahaba taipingensis* was collected from Dongguan offshore water, Guangdong, China. A piece of muscle tissue excised from the individual was preserved in 95% ethanol for DNA extraction. Total genomic DNA from muscle tissue was extracted with a standard phenol/chloroform procedure followed by ethanol precipitation and kept at 4 °C for subsequent use.

### Mitochondrial DNA amplification

The complete *Bahaba taipingensis* mitogenome was amplified using a long-PCR technique (Miya et al. 1999). Six sets of primers (Table 1) were designed based on multiple alignments of the conserved region of the complete mitochondrial DNA sequences of other Sciaenidae fishes: *Larimichthys crocea* (EU339149), *Collichthys niveatus* (JN678726), *Collichthys lucida* (JN857362), *Larimichthys polyactis* (FJ618559), *Miichthys miiuy* (HM447240) and *Pennahia argentata* (HQ890946), as well as previously determined, partial sequences of the 16S rRNA, Cyt b, COI genes and control region. Subsequent sequencing was accomplished by primer walking method. After the sequencing of these fragments, 31 normal PCR primer sets were designed using Premier 5.0 (Primer Biosoft International) to obtain contiguous, overlapping segments of the entire mitogenome. It was necessary that every two contiguous segments overlapped by at least 50 bp to ascertain the accuracy of sequencing.

**Table 1.** Primers used to amplify mtDNA of the *Bahaba taipingensis*.

Segment	Primer code	Nucleotide sequence(5'-3')	Expected product length	Annealling temperature
A	H16396-F	TGAGATCACTAACACTCCTGTA	3064bp	57 °C
	H2080-R	GTGACCATGAGTTTAACGG		
B	H2004-F	CGCCTGTTTAACAAAAACAT	4174bp	58 °C
	H6194-R	TAGACTTCTGGGTGGCCAAAGAATCA		
C	H6108-F	CAATGCTTCTAACAGACCG	3388bp	57 °C
	H9516-R	CAAGACCCGGTGATTGGAA		
D	H9428-F	TTGGCTCTACATTCCTAGC	3554bp	57 °C
	H12002-R	TAGGCTAGGAGGAAGAAGA		
E	H11932-F	CTCTTGGTGCAAATCCAAG	2471bp	56 °C
	H14423-R	AGTGCGTCGTTAGCGATTT		
F	H14326-F	AGGACTCTAACCAGGACTA	2181bp	56 °C
	H27-R	CATCTTAACATCTTCAGTGT		

All PCRs were performed in a Takara thermal cycler. Takara Ex-Taq and LA-Taq polymerase (Takara Biomedical) were used for normal and long-PCR reactions, respectively. Long-PCR reactions were carried out in 25 µl reaction mixture containing 15.25 µl of sterile distilled H_2_O, 2.5 µl of LA-Buffer, 4 µl of dNTP, 1 µl of each primer (5 µM), 0.25 µl of LA-Taq polymerase (1 unit/µl, Takara), and 1 µl of DNA template. The long-PCR reactions consisted of an initial denaturing step at 94 °C for 2 min, followed by 30 cycles of denaturing at 94 °C for 30 s, annealing at about 57 °C for 3 min and a final extension at 72 °C for 15 min. The normal PCR was performed following the standard procedure. Negative controls were included in all PCR amplifications to confirm the absence of contaminants. PCR products were cleaned by adding 0.45 µl of Shrimp Alkaline Phosphatase (Biotech Pharmacon), 0.9 µl of Exonuclease I (GE Healthcare) and 1.65 µl of sterile distilled H_2_O to 9 µL of PCR product and incubating at 37 °C for 30 min and 80 °C for 20 min. The purified product was then sequenced on ABI Prism 3730 (Applied Biosystems) from both strands with the same primers as those used for PCRs.

### Sequence editing and analysis

Sequence trace files were corrected and aligned with the DNAstar 5.0 software package (DNAstar, Inc.,Wisconsin, USA). The locations of 13 protein-coding genes and 2 rRNA genes were determined by their similarity to published mitogenomes of other Sciaenidae species as shown in Table 2, whereas the tRNA genes were identified using the program tRNAscan-SE 1.21 (Lowe et al. 1997). Some tRNA genes, e.g. tRNA-Ser (AGY) that could not be found by the tRNAscan-SE, were identified by their secondary structure and their position in the mitogenome (Zhang et al. 2009).

**Table 2.** Fish species analyzed in this study.

Species	Length/bp	GenBank accession no.
Family Sciaenidae		
Subfamly Pseudosciaeniae		
*Larimichthys crocea*	16466	EU339149
*Larimichthys polyactis*	16470	FJ618559
*Collichthys lucida*	16451	JN857362
*Collichthys niveatus*	16450	JN678726
*Miichthys miiuy*	16493	HM447240
subfamly Argyrosominae		
*Pennahia argentata* (China)	16485	HQ890946
*Pennahia argentata* (Japan)	16486	KC545800
*Nibea albiflora*	16499	HQ890947
*Nibea coibor*	16509	KM373207
subfamly Sciaeninae		
*Dendrophysa russelii*	16626	JQ728562
family Haemulidae		
*Parapristipoma trilineatum*	16546	NC009857

The structure of the control region and its conserves motifs were identified by making a comparison with homologous sequences of reported teleost (Lee et al. 1995; Cui et al. 2009; Cheng et al. 2010). The proposed secondary structure of the putative O_L_ was analyzed with the program Mfold v.3.2 with default setting (Zuker 2003) and visualized using RNAViz (De Rijk and De Wachter 1997).

### Phylogenetic analyses

To clarify the phylogenetic position of *Bahaba taipingensis* within the family Sciaenidae, the complete mitogenome sequences of 9 fish species with 10 complete mitogenome sequences in Sciaenidae (Table 2) were incorporated together with the presently obtained mitogenome sequence of *Bahaba taipingensis* for phylogenetic analysis. In addition, possible close outgroups in Percoidei (Table 2) were chosen to root phylogenetic trees (Boger and Kritsky 2003). Sequences were aligned using Clustal W (Thompson et al. 1994), and adjustments were made manually. Phylogenetic analyses were based on the concatenated sequences of 12 protein-coding genes and 2 rRNA. The ND6 gene was excluded because of its heterogeneous base composition and consistently poor performance in phylogenetic analysis (Miya et al. 2003). For protein-coding genes, all stop codons were excluded from the analysis. The possible bias of substitution saturation at each codon position of protein-coding genes and 2 rRNA genes was investigated using DAMBE v.4.5.57 (Xia et al. 2001), and the results suggested that the third codons position were saturated both for transitions and transversions in the plot against with pairwise sequence divergence. Finally, unambiguously aligned sequences were 3630, 3630, 2728 nucleotide positions from first and second codon position of 12 protein-coding genes, 2 rRNA genes, respectively, and thus a total of 9988 bp positions were utilized for phylogenetic analysis.

Two different methods, Bayesian inference (BI) and maximum likelihood (ML), were used to construct the phylogenetic tree. Three partitions (first and second codon positions of protein-coding genes, 2 rRNA genes) were set in the combined data set for partitioned Bayesian analyses using MrBayes 3.1.2 (Ronquist et al. 2003), which allowed different substitution models in individual partitions. Markov Chain Monte Carlo (MCMC) Bayesian analyses were undertaken with MrBayes 3.1.2 setting for the best-fit model of nucleotide evolution selected by Hierarchical Likelihood Ratio Tests (hLRTs) in MrModeltest version 2.3 (Posada et al. 2004). Four Markov chains (one cold and three heated) were used in each of two simultaneous runs starting from different random trees. Analyses were run for 1,000,000 generations, sampled every 100 generations to assess convergence. The distribution of log-likelihood scores was examined to determine stationarity for each search and to determine if extra runs were required to achieve convergence in log likelihoods searches. We discarded initial trees with non-stationary log-likelihood values as part of a burn-in procedure, and combined the remaining trees that resulted in convergent log-likelihood scores from both independent searches. These trees were used to construct a 50% majority rule consensus tree.

Maximum likelihood analysis (ML) was performed in PAUP 4.0 (Swofford 2000), and the GTR+I+G (I=0.45, G=0.88) model of DNA substitution for the analysis was assessed by Modeltest version 3.7 (Posada and Crandall, 1998). The ML analysis was performed with random sequence addition replicates. Heuristic search was undertaken using 10 random addition sequence starting trees and tree bisection reconnection (TBR) branch swapping. The confidence level (Felsenstein, 1985) at each branch was evaluated by performing bootstrapping (BP) with 100 replicates in ML analysis.

## Results and discussion

### Mitochondrial genomic structure

The complete mitogenome of *Bahaba taipingensis* was sequenced to be 16500 bp which consisted of 13 typical vertebrate protein-coding genes, 22 tRNA genes, 2 rRNA genes, and 1 putative control region (CR, Table 3). It had been submitted to GenBank with accession number JX232404. The mitogenome of *Bahaba taipingensis* had substantially similar patterns on mitogenome structural organization with other vertebrates (Anderson et al. 1981; Miya et al. 1999; Cui et al. 2009). The encoding genes of mitogenome were located on H-strand with the exception of ND6 and 8 tRNA genes that were transcribed from L-strand (Table 3). All genes from *Bahaba taipingensis* mitogenome were similar in size to most Perciformes species (Kim et al. 2004; Mabuchi et al. 2007; Cui et al. 2009; Cheng et al. 2011a; Cheng et al. 2012a) and the presence length of control region assumed variation in size, because they were prone to undergo the insertion/deletion events in the sequences (Sbisa et al. 1997).

**Table 3.** Characteristics of the mitochondrial genome of *Bahaba taipingensis*.

Gene	Position		Size(bp)	Amino acid	Condon Initiation	Stop	Intergenic nucleotide	Stand
	From	To	Nucleotide					
tRN^APh^e	1	68	68				0	H
12S rRNA	69	1017	949				0	H
tRNA^Val^	1018	1090	73				0	H
16S rRNA	1091	2792	1702				0	H
tRNA^Leu^(UUR)	2793	2866	74				0	H
ND1	2867	3841	975	324	ATG	TAG	4	H
tRNA^Ile^	3846	3915	70				-1	H
tRNA^Gln^	3915	3985	71				-1	L
tRN^AMe^t	3985	4054	69				0	H
ND2	4055	5099	1046	328	ATG	TA	0	H
tRNA^Trp^	5100	5170	71				1	L
tRNA^Ala^	5172	5240	69				2	L
tRNA^Asn^	5243	5315	73				37	L
tRNA^Cys^	5353	5418	66				0	L
tRNA^Tyr^	5419	5488	70				1	L
COI	5490	7046	1557	518	ATG	AGA	-5	H
tRNA^Ser^(UCN)	7042	7112	71				3	L
tRNA^Asp^	7116	7184	69				8	H
COII	7193	7883	691	230	ATG	T	0	H
tRNA^Lys^	7884	7957	74				1	H
ATPase8	7959	8126	168	55	ATG	TAA	-10	H
ATPase6	8117	8799	683	227	ATG	TA	0	H
COIII	8800	9584	785	261	ATG	TA	0	H
tRNA^Gly^	9585	9655	71				0	H
ND3	9656	10005	349	118	ATG	T	0	H
tRNA^Arg^	10005	10073	69				0	H
ND4L	10074	10370	297	98	ATG	TAA	-7	H
ND4	10364	11744	1381	460	ATG	T	0	H
tRNA^His^	11745	11813	69				0	H
tRNA^Ser^(AGY)	11814	11880	67				5	H
tRNA^Leu^(CUN)	11886	11958	73				0	H
ND5	11959	13797	1839	612	ATG	TAG	4	H
ND6	13794	14315	522	173	ATG	TAA	0	L
tRNA^Glu^	14316	14384	69				4	L
Cytb	14389	15529	1141	380	ATG	T	0	H
tRNA^Thr^	15530	15601	72				3	H
tRNA^Pro^	15605	15674	70				0	L
Control Region	15675	16500	826					H

The overall base composition of the *Bahaba taipingensis* mitogenome was estimated to be 28.2% for A, 31.1% for C, 16.2% for G, and 24.6% for T (Table 4), respectively, indicating an obvious antiguanine bias. Furthermore, the G content of all protein-coding genes presents obviously lower just as found in other bony fishes (Miya et al. 2003; Mabuchi et al. 2007). The most remarkable character of metazoan mitogenomes is the strand-specific bias in nucleotide composition (Reyes et al. 1998; Hassanin et al. 2005), which can be measured as GC-skew (G%-C%)/(G%+C%) and AT-skew (A%-T%)/(A%+T%), respectively (Perna et al. 1995). The overall GC- and AT-skews of the H-strand of *Bahaba taipingensis* mitogenome were -0.328 and 0.047, respectively, indicating a strand compositional bias characterized by a strong excess of C over G nucleotides and a slight excess of A over T nucleotides on the H-strand.

**Table 4.** Base composition for protein-coding, tRNA, and rRNA genes of *Bahaba taipingensis* mitogenome.

Gene/regon	Base composition(%)				A+T	number
	T	C	A	G		
ND1	25.9	35.3	24.5	14.3	50.4	975
ND2	24.6	38.1	25.6	11.7	50.2	1046
ND3	26.4	38.1	20.9	14.6	47.3	349
ND4	24.6	35	26.1	14.3	50.7	1381
ND4L	25.6	38.7	21.9	13.8	47.5	297
ND5	26.2	33.4	28.3	12.1	54.5	1839
ND6	12.3	35.4	38.3	14	50.6	522
COI	29.2	28.7	23	19.1	52.2	1557
COII	27.1	28.9	28.5	15.5	55.6	691
COIII	28.3	31.2	23.6	16.9	51.9	785
ATP6	25.2	38.4	23.4	13	48.6	683
ATP8	23.2	33.3	32.8	10.7	56	168
Cytb	26.8	35	24	14.2	50.8	1141
Protein coding						
1st	29.1	30.6	24	16.3	53.1	3630
2nd	21.7	35.1	26.9	16.3	48.6	3630
3rd	28.3	36.2	24.7	10.8	53	3630
Total	26.4	34	25.2	14.4	51.6	10890
tRNA	27.1	22.6	27.4	23.9	54.5	1553
rRNA	20.8	26.7	32.2	20.3	53	2651
D-loop	30.4	22.8	31.6	15.2	62	826
Overall	25.1	31.4	27.6	15.9	52.7	16500

### Protein-coding genes

The *Bahaba taipingensis* genome contained 13 protein-coding genes encoded on the H-strand excluding ND6 gene that was oriented to L-strand. The 13 protein-coding genes were total 11,436 bp in size, accounting for 69.15% of the whole mitogenome. All protein-coding genes initiated with an ATG codon, just as in most vertebrates. Three open reading frames (ATP8, ND4L and ND6) of *Bahaba taipingensis* ended with TAA, two open reading frames (ND1 and ND5) with TAG, and one open reading frames (COI) with AGA. The remainder used incomplete stop codons, either TA (ND2, ATP6 and COIII) or T (COII, ND3, ND4 and Cytb), probably completed by post-transcriptional polyadenylation (Ojala et al. 1981). It should be noted that these genes (ND4L with ND4, ATP8 with ATP6 and COI with tRNA^Ser^(UUR)) could complete their stopped codons within the overlapping portion of the next genes.

Nucleotide composition and codon using frequencies were calculated from a concatenated sequence of all protein-coding genes on the H-strand, except for ND6 on the L-strand. The base composition of protein-coding genes revealed weak bias against G (14.4%), especially at third codon positions (10.8%, Table 4). For all protein genes, C was the most frequent nucleotide at the first and third positions whereas T was most frequent at the second position as found in other bony fishes (Oh et al. 2007).

### Ribosomal and transfer RNA genes

Like other mitochondrial genomes (Zardoya et al. 1995; Inoue et al. 2000), twenty-two tRNA genes were identified. The tRNA genes were interspersed among the mitochondrial genome and ranged in size from 66 to 74 bp (Table 3). They showed the typical gene arrangement as found in most vertebrates. Fourteen tRNA genes were transcribed on the H-strand, whereas the remaining eight tRNA genes were oriented on the L-strand (Table 3). These tRNA genes were predicted capable of folding into typical cloverleaf secondary structures with normal base pairing. The *Bahaba taipingensis* mitogenome also contained a small subunit of rRNA (12S rRNA) and a large subunit of rRNA (16S rRNA) as in other bony fishes (Zardoya et al. 1995; Inoue et al. 2000), which were 947 bp and 1684 bp in length, respectively. As in other vertebrate genomes, these genes were located between the tRNA^Phe^ and tRNA^Val^ genes and between tRNA^Val^ and tRNA^Leu^(UUR) genes, respectively.

### Non-coding regions

As shown in Table 3, there were non-coding intergenic spacers from 1 to 8 bp observed in *Bahaba taipingensis*, spanning the contiguous genes apart from O_L_ and control region. Furthermore, mitochondrial intergenic spacers were a total of 36 bp in eleven different locations.

**Figure 1.**
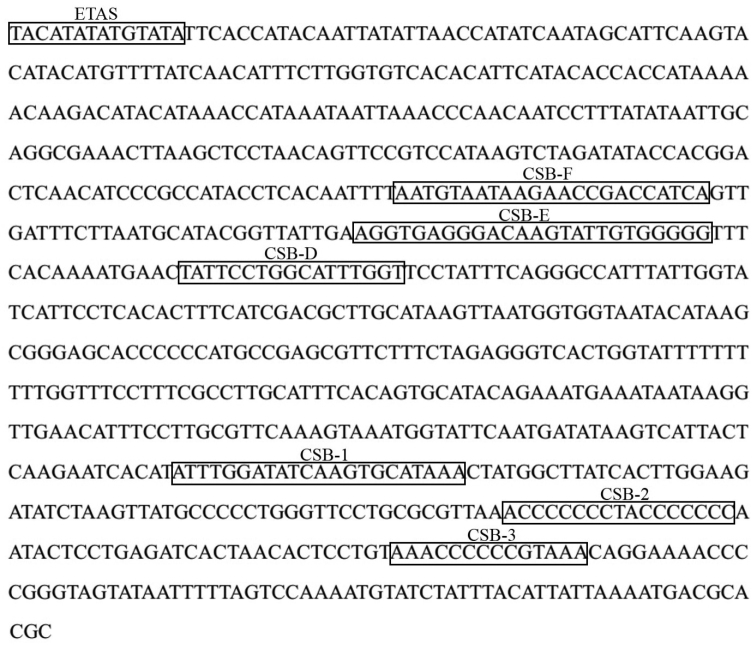
The structure of control region about *Bahaba taipingensis*.

As in most vertebrates, the major non-coding region in *Bahaba taipingensis* mitochondrial genome was located between tRNA-Pro and tRNA-Phe. It was determined to be 826 bp in length, longer than other reported Sciaenidae species, and it had an overall base composition that was rich in A and T (A+T=62.0%). By comparing with the recognition sites in some reported fishes (Lee et al. 1995; Cui et al. 2009; Cheng et al. 2010; Cheng et al. 2011a; Cheng et al. 2012a), three domains were detected in *Bahaba taipingensis*, namely, the termination associated sequence domain (ETAS), the central conserved sequence block domain (CSB-D, CSB-E and CSB-F) and the conserved sequence block domain (CSB-1, CSB-2 and CSB-3) (Figure 1). The ETAS was thought to act as a signal for the termination of H-strand elongation (Clayton 1991), and this domain was a hypervariable domain that might be useful for population genetic analyses. Furthermore, the motif sequence of ETAS was TACATAT with one palindromic sequence ATGTATA. The control region of mammals contained five blocks (CSB-B, CSB-C, CSB-D, CSB-E and CSB-F) in central conserved sequence blocks, however, only CSB-D, CSB-E and CSB-F were mostly detected in fishes (Brought et al. 1994; Lee et al. 1995). In this study, all these three motifs were identified in the central domain in accordance with *Miichthys miiuy* (Cheng et al. 2010), and *Nibea albiflora* (Cheng et al. 2011b) within Sciaenidae, which was not detected in four other species of Pseudosciaeniae (Cui et al. 2009; Cheng et al. 2011a; Cheng et al. 2012a; Cheng et al. 2012b). In addition, the consensus sequence of CSB-F was ATGTAATAAGAACCGACCAT, which distinguished the central conserved sequence block domain from the termination associated sequence domain. CSB-E was located downstream of CSB-F, whose consensus sequence was AGGGACAAGTATTGTGGGGG, characterized by the box GTGGGG. CSB-E was followed by CSB-D with its consensus sequence TATTCCTGGCATTTGGT. Generally, these key sequences were highly conserved and easily recognized. Three conserved sequence blocks (CSB-1, CSB-2 and CSB-3) were determined in the conserved sequence block domain which was thought to be involved in positioning RNA polymerase both for transcription and for priming replication (Shadel et al. 1997). Moreover, the critical central conserved sequences of CSB-1, CSB-2, and CSB-3 were ATTTGGATATCAAGTGCATAAA, ACCCCCCCTACCCCCCC, and AAACCCCCCGTAAA, respectively.

**Figure 2.**
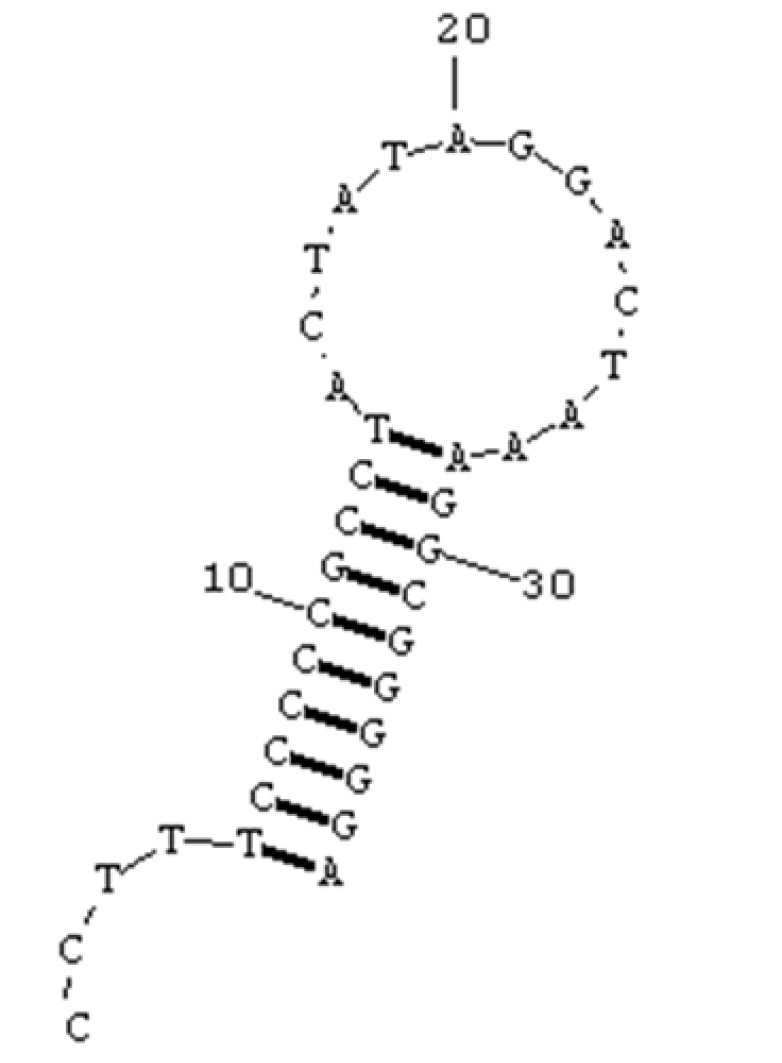
Potential secondary structure of the origin of L-strand replication (O_L_) of *Bahaba taipingensis* mtDNA.

The additional non-coding region, the putative origin of L-strand replication (O_L_), was located in a cluster of five tRNA genes (the WANCY region) between the tRNA-Asn and tRNA-Cys genes, almost identical with other Sciaenidae fishes. The putative O_L _could form a stable stem-loop secondary structure with 20 bp in the stem and 13 bp in the loop (Figure 2), which was 37 bp in length (CCTTTCCCCCGCCTACTATAGGACTAAAGGCGGGGGA). Furthermore, the conserved stem-loop structures in mitochondrial genomes was thought to play an importance role in conjunction with the origin of mtDNA replication.

### Phylogenetic analyses within family Sciaenidae

**Figure 3.**
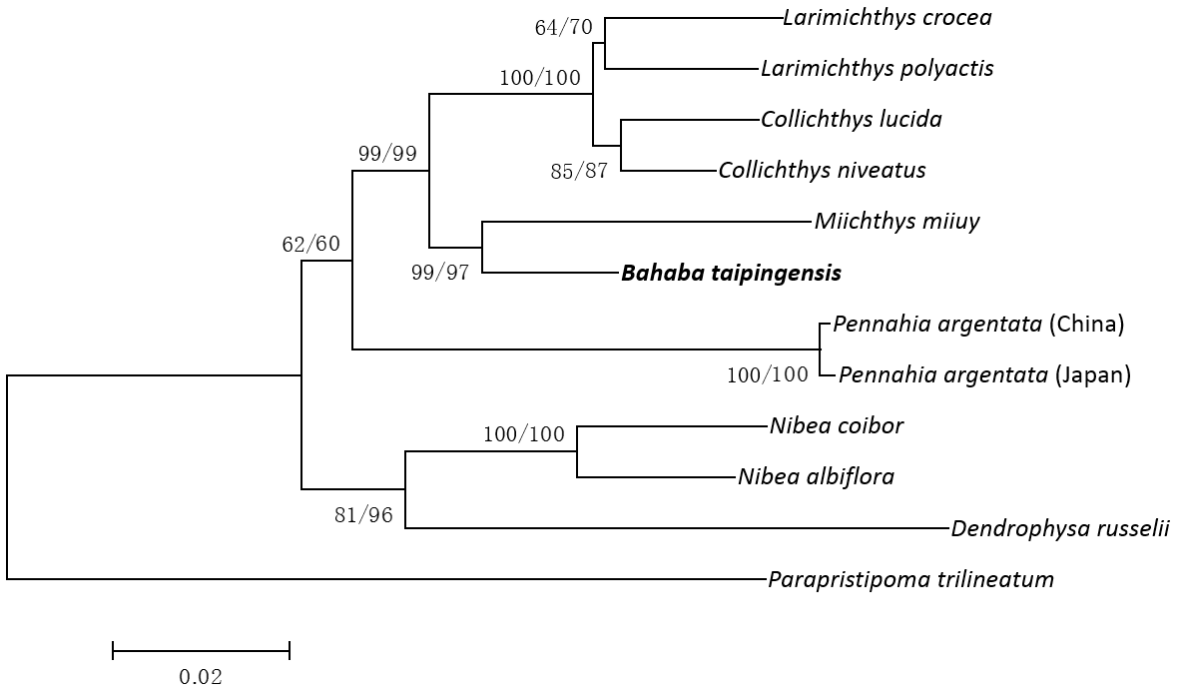
Phylogenetic relationships among Sciaenidae species based on the combined 9988 bp nucleotide positions. The posterior probability value of BI analyses and bootstrap support values of ML analyses (in the order: BI, ML) are indicated near the branches.

The phylogenetic trees (the 50% majority-rule consensus tree is shown in Figure 3) were highly coincident regardless of the analytic method used, and were statistically supported by high posterior probability and intermediate bootstrap values. This phylogenetic analysis represented the first investigation of relationships of *Bahaba taipingensis* within the Sciaenidae based on the whole mitogenome. In our analysis, *Bahaba taipingensis* was found to be more closely related to Pseudosciaeniae (*Collichthys*, *Larimichthys* and *Miichthys*) than to *Pennahia* and *Nibea*, the latter of which was suggested by morphological topology (Zhu et al. 1963; Cheng et al. 1987) and previous molecular study (He et al. 2012). However, phylogenetic analyses showed that *Miichthys* could not be merged into the *Collichthys*–*Larimichthys* clade. On the contrary, *Miichthys* and *Bahaba* formed an independent clade well supported by high posterior probability value, and this clade formed the sister group of the *Collichthys*–*Larimichthys* clade. Therefore, the relationship between *Miichthys* and Pseudosciaeniae deservesd to be further studied. The proposed phylogenetic position of *Bahaba taipingensis* within the Sciaenidae based on the findings of the present study should be accepted with caution due to limited taxon sampling. However, the phylogenetic relationship within the Sciaenidae remains to be resolved, and it is necessary to make further analysis based on more molecular information and extensive taxon sampling.
